# Patterns of Lisdexamfetamine Dimesylate Use in Children, Adolescents, and Adults with Attention-Deficit/Hyperactivity Disorder in Europe

**DOI:** 10.1089/cap.2019.0173

**Published:** 2020-08-28

**Authors:** Csaba Siffel, Matthew Page, Tricia Maxwell, Barbara Thun, Nikolaus Kolb, Mats Rosenlund, Dorothea von Bredow, Jacco Keja

**Affiliations:** ^1^ Global Evidence and Outcomes, Data Sciences Institute, Shire, a Takeda Company, Lexington, Massachusetts, USA.; ^2^ College of Allied Health Sciences, Augusta University, Augusta, Georgia, USA.; ^3^ Marketed Products Group, Chief Medical Office, Shire, a Takeda Company, Cambridge, Massachusetts, USA.; ^4^ Real World Solutions, IQVIA, Munich, Germany.; ^5^ Real World Solutions, IQVIA, Solna, Sweden.; ^6^ Department of Learning, Informatics, Management and Ethics (LIME), Karolinska Institutet, Stockholm, Sweden.; ^7^ Real World Solutions, IQVIA, Paris, France.

**Keywords:** drug utilization, treatment patterns, lisdexamfetamine dimesylate, ADHD

## Abstract

***Objectives:*** Lisdexamfetamine dimesylate (LDX) is approved in some European countries for the second-line treatment of attention-deficit/hyperactivity disorder (ADHD) in children and adolescents when response to previous methylphenidate (MPH) treatment is considered clinically inadequate, and as a first-line treatment in adults. Limited evidence exists on the real-world use of LDX across Europe. This retrospective study evaluated LDX drug utilization patterns from eight European countries for up to 5 years.

***Methods:*** Data were collected from national registries (Denmark, Finland, Norway, Sweden), electronic medical records (Germany, Spain, United Kingdom), and prescription databases (Switzerland) in eight European countries. Patients were included if they were prescribed LDX at least once since the LDX launch date in each country. Demographic and clinical characteristics, and LDX prescription data included patient age and gender, a recorded diagnosis of ADHD, the number of prescriptions per participant, previous MPH prescription recorded, average daily dose, treatment persistence, discontinuation, and switching of medications.

***Results:*** Overall, information for 59,292 patients (437,272 LDX prescriptions) was analyzed. Most patients were male (58.1%–84.3%) and fewer than 1% were under 6 years of age. Extensive use of LDX in adults was observed in four countries (Denmark, Finland, Norway, and Sweden), including countries where LDX was not approved for this age group. Most patients had a recorded diagnosis of ADHD (61.9%–95.4%). The mean number of prescriptions per patient ranged from 5.4 to 10.0. At least 79.6% of patients with ADHD had a recorded previous MPH prescription. Mean duration of LDX exposure ranged from 233.1 to 410.8 days. The average daily dose of LDX was ≤70 mg/day for most patients (79.4%–99.7%). The 5-year discontinuation rate ranged from 22.8% to 70.6% and was below 40% for most countries. The proportion of patients switching from LDX to other medications was ≤33.8.

***Conclusions:*** This study provides the first long-term, real-world information related to LDX use by children, adolescents, and adults in Europe in the 5 years since its first launch in the region. Most LDX prescriptions fulfilled label requirements regarding a recorded diagnosis of ADHD before treatment initiation, previous MPH use, and an average daily dose of ≤70 mg/day. LDX was largely prescribed within the indicated age range, although adult use of LDX was high in some countries where LDX is not approved for this population.

## Introduction

Attention-deficit/hyperactivity disorder (ADHD) is a common neurodevelopmental disorder with an estimated worldwide prevalence in children and adolescents of ∼5% (Polanczyk et al. 2007). ADHD often persists beyond childhood, with a reported prevalence in adults in the range of 3%–5% across different countries (Fayyad et al. 2007; Simon et al. 2009). In Europe, methylphenidate (MPH) is the only psychostimulant licenced and recommended by guidelines as first-line therapy for the treatment of children and adolescents with ADHD (National Institute for Health and Care Excellence [NICE] 2018). In North America, amphetamines (AMPs) are recommended additionally as a first-line treatment (AAP 2011; CADDRA 2018). In Europe and North America, lisdexamfetamine dimesylate (LDX) and MPH are both licenced and recommended to be used in first-line treatment of adults with ADHD (AAP 2011; CADDRA 2018; NICE 2018). Evidence from a recent network meta-analysis supports MPH (in children and adolescents) and AMPs (in adults) as the preferred first choice for short-term pharmacological treatment of ADHD (Cortese et al. 2018). This concurs partly with the NICE (2018) recommendations.

The efficacy of the long-acting AMP prodrug LDX in relieving the symptoms of ADHD has been demonstrated in a series of pivotal randomized controlled trials in North America and Europe (Biederman et al. 2007; Adler et al. 2008; Findling et al. 2011; Coghill et al. 2013). Evidence from trials of at least 12-month duration indicates that the safety and tolerability profile of LDX is similar to those of other stimulants in people with ADHD (Findling et al. 2008; Weisler et al. 2009; Coghill et al. 2014).

LDX was first approved in the United States for the treatment of ADHD in children and adolescents in 2007 (Vyvanse Shire US, Inc. 2016), followed in 2013 in certain European countries for the treatment of children and adolescents with ADHD whose response to previous MPH treatment was considered clinically inadequate (Elvanse: Shire Pharmaceuticals Ltd. 2016b). Since then, LDX has also been approved for the treatment of adults with ADHD in several European countries, including the treatment of adults not older than 55 years in Switzerland (2014) and all adults in Denmark, Sweden, and the United Kingdom (2015) (Elvanse Adult: Shire Pharmaceuticals Ltd 2016a). The NICE guidelines in the United Kingdom include LDX in the recommendation for first-line pharmacological treatment of adult ADHD (NICE 2018).

However, there is little evidence of how the treatment is being used in terms of patient characteristics and prescribing patterns in the real world. Drug utilization studies are important to understand the use of a treatment in routine clinical practice. Previous drug utilization studies have shown that treatment adherence and persistence are generally greater for long-acting stimulants than for short-acting stimulants, and that adherence to, and persistence with, AMP treatment is greater than for MPH treatment (Christensen et al. 2010; Lawson et al. 2012). Since its approval in Europe in 2013, published information related to the use of LDX in the real-world setting has been lacking. The objective of this retrospective, European safety study was to evaluate drug utilization patterns and monitor off-label use of LDX in the 5 years since LDX was first launched in the region as part of Shire's ongoing pharmacovigilance programme for LDX; this study was not intended to compare the safety of LDX with other drugs for the treatment of ADHD. This includes the characterization of patients, description of LDX prescribing patterns among physicians and usage patterns among patients, and potential off-label use.

## Methods

### Ethics

This study was conducted in accordance with current applicable international and national regulations and ethical requirements. The study was approved by the relevant local governing bodies in each country.

### Study design and database use

This drug utilization study was conducted to investigate the use of LDX since its launch in Denmark, Germany, Ireland, Sweden, and the United Kingdom in 2013, with Finland, Norway, Spain, and Switzerland following in 2014. Registries and databases from all these countries except Ireland were used to analyze LDX drug use (Table 1); Ireland was excluded owing to limited data availability.

**Table 1.** Observation Period

	Denmark	Finland	Germany	Norway	Spain	Sweden	Switzerland	United Kingdom
LDX launch date	March 2013	September 2014	June 2013	September 2014	May 2014	September 2013	August 2014	March 2013
Approval for adults	2015	N/A	N/A	N/A	N/A	2015	2014 (adults ≤55 years)	2015
Observation period	March 2013–December 2016	September 2014–December 2016	June 2013–December 2017	September 2014–December 2016	May 2014–December 2017	September 2013–December 2016	August 2014–December 2017	March 2013–December 2017
Database	National registry	National registry	DA	National registry	LPD	National registry	IQVIA PPP/IQVIA SDPP	CPRD

CPRD, Clinical Practice Research Datalink; DA, disease analyzer; IQVIA PPP, IQVIA pharmacy prescription panel; IQVIA SDPP, IQVIA self-dispensing prescription panel; LDX, lisdexamfetamine dimesylate; LPD, Longitudinal Patient Database; N/A, not applicable.

The registries and databases used for this study included longitudinal electronic medical record (EMR) databases covering patient and prescription data for Germany (IQVIA Disease Analyzer [DA]), Spain (IQVIA Longitudinal Patient Database), and the United Kingdom (Clinical Practice Research Datalink [CPRD]). For the DA and CPRD databases, only physician offices that entered data into the database every month for the entire observation period were considered. For Denmark, Finland, Norway, and Sweden, data from the national registries for these countries were used; these registries are longitudinal databases providing almost complete coverage of prescribed and dispensed medications and diagnoses from in- and outpatient care for the entire population of each country. Prescription databases using pharmacy retail data were used for Switzerland (IQVIA Pharmacy Prescription Panel [PPP] and IQVIA Self-Dispensing Prescription Panel [SDPP]).

All patients who had been prescribed LDX at least once during the study period from March 2013 (first European launch) to December 2017 were included in the study. As detailed in Table 1, exact observation periods per country were dependent on LDX launch dates and reporting dates, ranging from 28 months in Finland and Norway to 58 months in the United Kingdom.

### LDX drug utilization analysis

Information captured included whether a diagnosis of ADHD was recorded in the database (this information was not available in the prescription databases in Switzerland), the number of prescriptions per patient, and the duration of exposure to LDX. Some variables, such as average daily dose, were not recorded in the databases and were calculated from available prescription data. Further treatment patterns were described in terms of treatment discontinuation and switching of medications. Discontinuation of LDX treatment was defined as not receiving a new prescription within 30 days following the end of the previous prescription period, without any evidence of switching to a different treatment. Switching was categorized as switching to LDX and switching from LDX. Switching from other medication to LDX was defined as a recorded LDX prescription within 30 days following the end of the previous prescription period of other ADHD medication. Switching from LDX to other medication was defined as a recorded prescription of any other ADHD medication within 30 days following the end of an LDX prescription period. Definitions of treatment patterns were in line with other pharmacoepidemiologic studies in the field of ADHD medications (Gajria et al. 2014; Greven et al. 2017).

For this study, the following were considered off-label use of LDX: (1) patients with no ADHD diagnosis recorded in the database before their first LDX prescription; (2) patients with no recorded previous MPH prescription; (3) children younger than 6 years at the time of prescription; and (4) patients with a prescribed dosage exceeding the recommended maximum daily dose of 70 mg/day. Adult (defined here as ≥19 years old) LDX use was considered off-label unless: (1) treatment continued from adolescence into adulthood; (2) patients were not older than 55 years in Switzerland; or (3) patients were prescribed LDX in Denmark, Sweden, or the United Kingdom in 2015 or later. The overall number of adult patients receiving LDX considered off-label from 2013 to 2016 was not available for Denmark, because the drug was not approved for use in adults in this country until 2015. Evaluation of off-label use in Switzerland was based on the IQVIA PPP data only, because most criteria could not be evaluated for IQVIA SDPP.

## Results

### Demographics

As detailed in Table 2, overall, information for 59,292 patients with 437,272 LDX prescriptions was collected through registries and databases in eight European countries during a maximum of 5 years since the initial approval of LDX in Europe in 2013. The majority of participants who received LDX were male (58.1%–84.3%; Table 2). The percentages of adults (≥19 years of age) among all patients receiving LDX were ∼8% in Spain, 9% in Germany, 19% in the United Kingdom, 24% in Finland, 46% in Norway, 47% in Denmark, 57% in Sweden, and 59% in Switzerland (Table 2).

**Table 2.** Participant Demographics

Category	Denmark**(*n = *8410)	Finland**(*n = *833)	Germany (*n = *3305)		Norway**(*n = *4796)	Spain (*n = *662)	Sweden**(*n = *37,241)	Switzerland (*n = *2806)		United Kingdom (*n = *1239)
Database	National registry	National registry	DA, PP (*n* = 1391)	DA, NPP (*n* = 1914)	National registry	LPD	National registry	IQVIA PPP (*n* = 1667)	IQVIA SDPP (*n* = 1139)	CPRD
Documented ADHD diagnosis, *n* (%)	5210 (62.0)	654 (78.5)	1327 (95.4)	1723 (90.0)	3479 (72.5)	410 (61.9)	32,818 (88.1)	N/A	N/A	1089 (87.9)
Prescriptions, *n*	74,608	5392	13,845	16,088	31,542	5272	278,049	N/A	N/A	12,476
Male, *n* (%)	5077 (60.4)	662 (79.5)	1172 (84.3)	1466 (76.6)	2888 (60.2)	527 (79.6)	21,631 (58.1)	1051 (63.0)	780 (68.5)	985 (79.5)
Age group (years), *n* (%)										
0–5	16 (0.2)	2 (0.2)	6 (0.4)	12 (0.6)	8 (0.2)	5 (0.8)	94 (0.3)	0 (0.0)	N/A	<6 (—)^a^
6–12	2047 (24.3)	463 (55.6)	876 (63.0)	1043 (54.5)	1461 (30.5)	258 (39.0)	6965 (18.7)	322 (19.3)	N/A	486 (39.2)
13–18	2426 (28.8)	170 (20.4)	498 (35.8)	694 (36.3)	1112 (23.2)	348 (52.6)	8918 (23.9)	361 (21.7)	N/A	516 (41.6)
19–25	1216 (14.5)	83 (10.0)	5 (0.4)	49 (2.6)	491 (10.2)	9 (1.4)	5559 (14.9)	265 (15.9)	N/A	103 (8.3)
>25	2705 (32.2)	115 (13.8)	3 (0.2)	116 (6.1)	1724 (35.9)	42 (6.3)	15,705 (42.2)	N/A	N/A	133 (10.7)
26–55	N/A	N/A	N/A	N/A	N/A	N/A	N/A	665 (39.9)	N/A	N/A
>55	N/A	N/A	N/A	N/A	N/A	N/A	N/A	54 (3.2)	N/A	N/A

^a^ Following CPRD policy, no exact numbers are included if *n* < 6 events.

ADHD, attention-deficit/hyperactivity disorder; CPRD, Clinical Practice Research Datalink; DA, disease analyzer; IQVIA PPP, IQVIA pharmacy prescription panel; IQVIA SDPP, IQVIA self-dispensing prescription panel; LPD, Longitudinal Patient Database; N/A, not applicable; NPP, neurologist/psychiatrist panel; PP, pediatrician panel.

### Treatment patterns

A diagnosis of ADHD was documented for most patients (61.9%–95.4%) (Table 2). At least 67.8% of patients in any given study country were considered repeat users, because they received more than one prescription of LDX during the study period (Table 3). The mean number of prescriptions per patient was lowest in the IQVIA PPP database in Switzerland (5.4) and highest in the United Kingdom CPRD and the German DA paediatrician panel databases (10.0 in both) (Table 3).

**Table 3.** Treatment Patterns

Category	Denmark (*n = *8410)	Finland (*n = *833)	Germany (*n = *3305)		Norway (*n = *4796)	Spain (*n = *662)	Sweden (*n = *37,241)	Switzerland (*n = *2806)		United Kingdom (*n = *1239)
Database	National registry	National registry	DA, PP (*n* = 1391)	DA, NPP (*n* = 1914)	National registry	LPD	National registry	IQVIA PPP (*n* = 1667)	IQVIA SDPP (*n* = 1139)	CPRD
Repeat users, *n* (%)	6927 (82.4)	671 (80.6)	1123 (80.7)	1515 (79.2)	3902 (81.4)	502 (75.8)	30,422 (81.7)	1160 (69.6)	772 (67.8)	1047 (84.5)
Prescriptions per participant*, n*										
Mean (SD)	8.9 (9.8)	6.5 (5.6)	10.0 (10.9)	8.4 (10.0)	6.6 (7.6)	8.0 (6.9)	7.5 (8.1)	5.4 (6.3)	7.3 (9.1)	10.0 (11.9)
Median (min; max)	6 (1; 127)	5 (1; 35)	6 (1; 60)	5 (1; 131)	4 (1; 161)	4 (1; 44)	5 (1; 145)	3 (1; 84)	3 (1; N/A)	6 (1; 183)
Duration of exposure, days										
Mean (SD)	247.3 (258.3)	272.7 (225.6)	410.8 (414.0)	389.1 (413.4)	233.1 (226.1)	338.0 (292.2)	306.5 (287.0)	267.9 (295.9)	N/A	354.2 (358.7)
Median (min; max)	152 (22; 1401)	218.2 (18; 851)	256 (15; 1654)	226 (15; 1661)	148 (2; 851)	184 (30; 1335)	212.0 (6; 1216)	132 (30; 1379)	N/A	226 (1; 1709)
Average daily dose,^a^ mg/day										
Mean (SD)	41.2 (15.4)	33.2 (14.8)	43.6 (14.7)	44.2 (15.3)	40.0 (16.0)	35.6 (7.5)	45.2 (16.2)	33.3 (16.4)	N/A	47.5 (16.0)
Median (min; max)	41.2 (0.8; 70.4)	30.0 (2.7; 70.0)	50 (10; 70)	50 (5.8; 70)	39 (1; 70)	30 (30; 70)	50.0 (2.9; 70.0)	31.6 (1; 70)	N/A	50 (10; 70)
Discontinued, *n* (%)^b^	1920 (22.8)	220 (26.4)	578 (41.6)	646 (33.8)	1888 (39.4)	203 (30.7)	13,912 (37.4)	624 (37.4)	804 (70.6)	778 (62.8)
Switched to LDX from other medication, *n* (%)^c^	6286 (74.7)	86 (10.3)	1047 (75.3)	1440 (75.2)	609 (12.7)	547 (82.6)	14,224 (38.2)	604 (36.2)	450 (39.5)	274 (22.1)
Switched from LDX to other medication, *n* (%)^d^	2502 (29.8)	89 (10.7)	241 (17.3)	430 (22.5)	545 (11.4)	224 (33.8)	4936 (13.3)	251 (15.1)	171 (15.0)	304 (24.5)
Adult prescription not continued from childhood/adolescent prescription, *n* (%)	Not available	207 (24.8)	7 (0.6)	99 (7.7)	2292 (47.8)	48 (7.5)	5370 (14.4)^e^	44 (3.8)^f^	Not available	27 (2.6)^e^

^a^ Average daily dose is calculated for prescriptions with dose recommendations ≤70 mg/day.

^b^ No prescription recorded for ≥30 days after the end of the previous prescription period with no evidence of switching to a different therapy.

^c^ Prescription of LDX within 30 days following the end of the last filled prescription period of ADHD medication other than LDX.

^d^ Prescription for an ADHD medication other than LDX within 30 days following the end of the last filled prescription period of LDX.

^e^ Patients who started LDX at age ≥18 years before label extension for adult use (January 2015).

^f^ Off-label use in Switzerland is described as age >55 years.

ADHD, attention-deficit/hyperactivity disorder; CPRD, Clinical Practice Research Datalink; DA, disease analyzer; IQVIA PPP, IQVIA pharmacy prescription panel; IQVIA SDPP, IQVIA self-dispensing prescription panel; LDX, lisdexamfetamine dimesylate; LPD, Longitudinal Patient Database; N/A, not applicable; NPP, neurologist/psychiatrist panel; PP, pediatrician panel; SD, standard deviation.

**FIG. 1.**
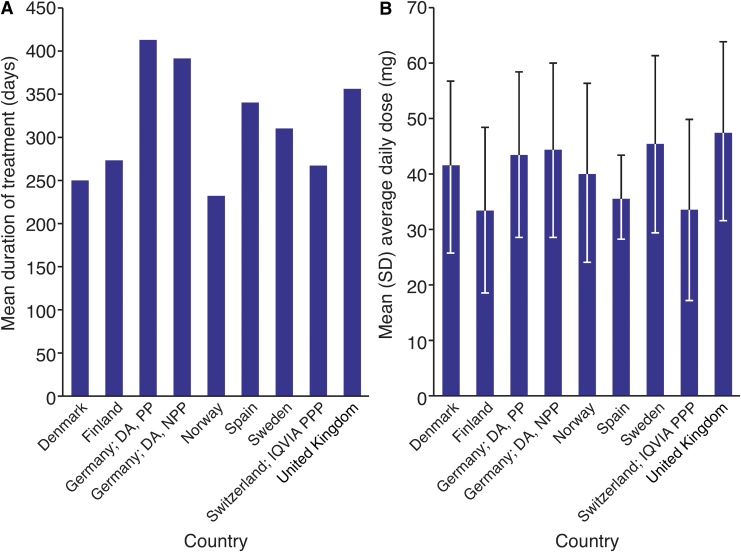
Treatment patterns of LDX. **(A)** Average duration (in days) of treatment with LDX across countries. **(B)** Average daily dose (calculated) of LDX across countries. DA, disease analyzer; IQVIA PPP, IQVIA pharmacy prescription panel; LDX, lisdexamfetamine dimesylate; NPP, neurologist/psychiatrist panel; PP, pediatrician panel; SD, standard deviation.

The mean duration of exposure to LDX across study countries ranged from 233.1 days (Norway) to 410.8 days (Germany) (Fig. 1A; Table 3). The calculated average daily dose of LDX was within the recommended range (30–70 mg/day) for most patients, with not more than 20.6% of patients in any given study country receiving >70 mg/day. The mean average daily dose for patients with prescriptions not more than 70 mg/day ranged from 33.2 to 47.5 mg (Fig. 1B; Table 3). No clear relationship between dosing and age group was observed (data not shown).

**FIG. 2.**
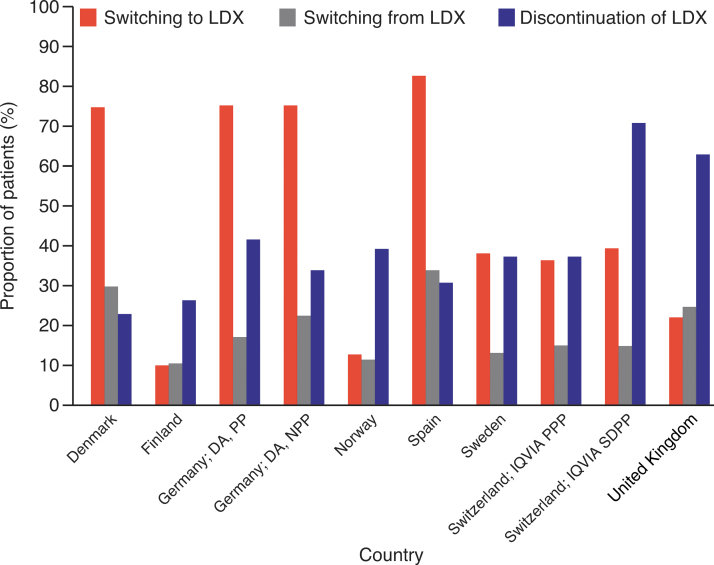
Discontinuation and switching patterns of treatment with LDX across countries. DA, disease analyzer; IQVIA PPP, IQVIA pharmacy prescription panel; IQVIA SDPP, IQVIA self-dispensing prescription panel; LDX, lisdexamfetamine dimesylate; NPP, neurologist/psychiatrist panel; PP, pediatrician panel.

The proportion of patients discontinuing treatment across study countries ranged from 22.8% (Denmark) to 70.6% (Switzerland). For most countries, the discontinuation rate was <40% for the cumulative observation period of up to 5 years (Fig. 2; Table 3). The proportion of patients who switched from LDX to other medications in study countries was not more than 33.8% (Fig. 2; Table 3).

Overall, for patients with an enrolment history of at least 365 days in databases in which the relevant data were available, not more than 20.4% recorded no prior use of MPH. A maximum of 43.9% of patients did not have a recorded ADHD diagnosis before receiving the first prescription of LDX in Denmark. Fewer than 1% of patients were younger than 6 years of age. Fewer than 2% of patients exceeded an average daily dose of >70 mg/day in Germany, Spain, and the United Kingdom; however, in Norway, a maximum of 20.6% of patients received an average daily dose >70 mg/day. The majority of patients with a potential average daily dose >70 mg/day were adult users.

The proportion of patients whose treatment was considered off label because they were adults at the time of prescription was <10% in Germany, Spain, Switzerland, and the United Kingdom, and 14.4% in Sweden. In Denmark, Sweden, Switzerland, and the United Kingdom, the overall proportion of patients considered off-label users decreased substantially after LDX was approved for adult use in these countries. In Finland and Norway, where adult use of LDX was not approved during the study period (approved in July 2017 and August 2017, respectively), the proportion of patients whose use was considered off label because they were adults was 24.8% and 47.8%, respectively.

## Discussion

This study provides the first long-term, real-world information related to the use of LDX by children, adolescents, and adults in Europe. The current study was conducted as part of a risk management plan commitment to investigate potential off-label use in a real-world setting. Most LDX prescriptions fulfilled label requirements regarding age (including adults in some countries), a recorded diagnosis of ADHD before treatment initiation, previous MPH use, and a calculated average daily dose of not more than 70 mg/day. Reasons for off-label LDX use included a lack of a recorded ADHD diagnosis in some countries, and adult prescriptions in countries where the use of LDX by adults was not approved at the start of the observation period.

The proportion of patients without a recorded diagnosis of ADHD before a prescription of LDX ranged from <10% (Germany) to >40% (Denmark). These values may represent the true off-label use of LDX in Europe; however, database records may underestimate the true extent of prior diagnoses, because the entry of diagnostic information in some national prescription databases is optional or can only be obtained by linking to other databases. Other databases may not capture diagnoses, or patients may have been seen outside the public sector and were thus not captured in the linked database.

Persistence of LDX treatment varied among countries, with a mean treatment duration ranging from just below 8 months to almost 14 months. These persistence data are generally higher than those reported for other psychostimulants, although different observation periods complicate their comparison (Marcus et al. 2005; Hodgkins et al. 2012; Gajria et al. 2014; Greven et al. 2017). Continued adequate ADHD symptom control is important, because untreated ADHD is often associated with social and academic difficulties, behavioural problems (such as substance abuse), delinquency, accidental injury, and poor economic, social, and emotional well-being (Shaw et al. 2012). A systematic literature review found that mean persistence was higher with long-acting stimulants and AMPs (∼250 days) than with short-acting stimulants and MPH, and that treatment duration was longer for stimulants than for nonstimulants (Gajria et al. 2014). In a United States study of 6-month follow-up, the mean persistence with LDX and mixed amphetamine salts (MAS extended release [XR]) was similar, and both were significantly higher than with atomoxetine (ATX) or osmotic-release oral system MPH (OROS-MPH) (Hodgkins et al. 2012).

In all countries, most patients were receiving less than the maximum approved LDX dose of 70 mg/day. Average daily doses in excess of that approved for ADHD pharmacotherapy may suggest that symptom control provided by the recommended dose range was inadequate. Patients receiving a dose >70 mg/day tended to be adults. Proportions of adult users and proportions of patients receiving >70 mg/day were highest in Nordic countries. In a recent 3-year study in adults in France, the calculated average daily dose of MPH exceeded the maximum approved dose according to European labels (Pauly et al. 2018). Two United States studies have shown that the calculated daily average consumption, relative to the prescribed dose, is generally lower for LDX than for OROS-MPH, MAS XR, or ATX (Hodgkins et al. 2012; Joseph et al. 2016).

It has previously been suggested that the likelihood of treatment discontinuation grows with increased duration of follow-up (Charach et al. 2004). It could be speculated that the long duration of follow-up for the United Kingdom, one of the countries in which LDX was first launched in Europe, may explain the high level of discontinuation reported for this country. However, Denmark had a similar follow-up period and substantially lower discontinuation than the United Kingdom. Furthermore, rates of switching from LDX were similar in Denmark and the United Kingdom, suggesting that other factors are involved. In an earlier (United States) study, the rate of discontinuation in adults was within the range observed in the present study, despite a shorter study duration (1 year). Discontinuation of LDX in the present study was somewhat lower than that reported for ATX (Joseph et al. 2016), whereas in another 1-year (United States) study, discontinuation of MPH in children was within the range seen in the present study (Faraone et al. 2007).

In some European countries, such as the United Kingdom, treatment guidelines generally recommend LDX for children and adolescents who were previously inadequately treated with MPH (NICE 2018). Reflecting this, prior MPH use was identified in at least 79.6% of patients within the databases in the present study. Similarly, the proportions of patients switching to LDX from other ADHD medications (e.g., ATX or MPH) were generally high. The lower proportions of patients switching in some countries than in others could be the result of long treatment gaps (>30 days), reported as discontinuation of previous treatment before receiving LDX. In some countries, prior MPH use is not required before prescribing other stimulants, including LDX, for example, for children and adolescents in Spain and for adults in the United Kingdom (GPC 2017; NICE 2018).

Most patients receiving LDX were within the indicated age range. Fewer than 1% of patients were below the lower bound of the approved age range for LDX (i.e., <6 years). For comparison, in an observational study of off-label use of ADHD medications in the United States, during 2012, for children aged 3–5 years, 1.7% of prescriptions for ADHD medications were written for LDX, while 18.8% were written for extended-release MPH (Panther et al. 2017). Treatment guidelines generally do not recommend pharmacological treatment for children younger than 6 years (Taylor et al. 2004; NICE 2018). A substantial number of adults (defined for the current analyses as aged ≥19 years) received prescriptions for LDX, which is approved for use in adults in Denmark, Sweden, Switzerland (≤55 years), and the United Kingdom. Adult use of LDX was also high in Finland and Norway, where LDX use is only approved when patients continued treatment into adulthood from adolescence. In the United States, LDX has been approved for use in adults since 2008; in 2013, 31.5% of adults with ADHD who were receiving long-acting ADHD medication as monotherapy were prescribed LDX. Off-label use of ADHD medication in adults was restricted to nonstimulants guanfacine and clonidine (Zhou et al. 2018).

### Strengths and limitations

Strengths of this drug utilization study include the large numbers of patients from eight European countries receiving LDX in the real-world setting, together with a long observation period of up to 5 years. The Nordic registries included here are mandatory nationwide registries with almost complete coverage of prescribed and dispensed medications and diagnosis, providing robust and reliable data for real-world evidence studies (Wettermark et al. 2013).

Differences between country-specific ADHD treatment guidelines may lead to high estimates of off-label use in some countries. For example, NICE guidelines in the United Kingdom recommend LDX as first-line pharmacological treatment in adults with ADHD without the need for prior MPH use (NICE 2018). In Spain, treatment guidelines do not require MPH to be used first-line before LDX prescriptions in children and adolescents. Hence, although the prescription of LDX for children and adolescents in Spain or for adults in the United Kingdom, with no record of prior MPH use, follow these countries' specific guidelines, this is still considered to be off label in the present analyses (GPC 2017; NICE 2018). The data analyzed were from early in the postauthorization lifecycle of LDX in Europe and drug utilization may change over time. Further studies will be required to establish whether this is the case.

The German, Spanish, and United Kingdom databases have known limitations associated with provider-sourced EMR databases (Becher et al. 2009). In Germany and, to some extent, in Spain, patients cannot be tracked across practices or specialties. Patients who seek care outside the EMR practice setting would not have these data recorded in the database. These EMR databases contain prescriptions written by the participating physicians, but do not contain actual prescription fills. Furthermore, treatment history is not available for patients who have changed from a previous provider or region that is not covered by the database, which could lead to underestimation of the number of patients with a diagnosis of ADHD, previous MPH prescriptions, or LDX prescriptions during childhood/adolescence. In addition, while the databases can provide information on prescriptions, they cannot confirm that the patients have actually taken the medication. Finally, calculation of the average daily dose does not account for treatment gaps, and might therefore result in underestimation of the average daily dose and the potential for patients receiving a daily dose above the approved dose. Differences between the databases used in this study limit direct comparisons between countries.

## Conclusions

Overall, most patients received LDX prescriptions within label requirements with regard to a recorded ADHD diagnosis, recorded prior MPH use, age, and recommended doses. The mean duration of LDX treatment was longer in this study (>233 days) than reported elsewhere for MPH, ATX, or LDX. Discontinuation rates were generally similar to those observed for psychostimulants in studies of much shorter duration.

## Clinical Significance

Drug utilization studies provide insights into treatment patterns and potential off-label use in a real-world setting. This study highlights that LDX use was generally within the label requirements based on age (including adults in some countries), a recorded diagnosis of ADHD before treatment initiation, previous MPH use, and a calculated average daily dose of not more than 70 mg/day.
